# Impact of a Multimedia Campaign to Increase Intention to Call 9-1-1 for Stroke Symptoms, Upstate New York, 2006-2007

**Published:** 2010-02-15

**Authors:** Janine M. Jurkowski, Dayna M. Maniccia, Deborah A. Spicer, Barbara A. Dennison

**Affiliations:** University at Albany School of Public Health, Department of Health Policy, Management, and Behavior; University at Albany School of Public Health, Albany, New York; New York State Department of Health, Albany, New York; New York State Department of Health, Albany, New York

## Abstract

**Introduction:**

Many people are not aware of stroke symptoms, the need for emergency care for those symptoms, and that calling 9-1-1 is recommended. The New York State Department of Health developed and implemented a multimedia campaign to increase stroke symptom awareness and awareness of the need to call 9-1-1.

**Methods:**

The evaluation of the campaign's impact was a pre/post intervention matched comparison design. A random-digit–dialed list-assisted telephone survey was administered to measure reach of the campaign and change in intention to seek emergency care for stroke by calling 9-1-1 in response to 4 signs or symptoms.

**Results:**

A larger proportion of respondents in the intervention region than in the comparison region reported seeing a stroke advertisement and reported the advertisement's message was to call 9-1-1. There was a significant increase between baseline and follow-up in intention to call 9-1-1 for the 4 stroke symptoms. These increases were greater in the intervention region than the comparison region. The differences between intervention and comparison groups in the increases in intention to call 9-1-1 ranged from 9% to 12% for specific stroke symptoms identified in oneself and from 4% to 12% for symptoms identified in another person.

**Conclusion:**

This multimedia campaign effectively increased intention to call 9-1-1 for stroke symptoms in the intervention region compared with a region matched for demographics and stroke rates. Multimedia campaigns are effective in increasing awareness of stroke symptoms and intention to immediately call 9-1-1.

## Introduction

Stroke is a significant cause of illness and death. In New York State, the stroke death rate is 34 per 100,000 people, and approximately 55,000 people per year are hospitalized for new or recurrent strokes (1,2). Current guidelines specify that treatment of acute ischemic stroke begin within 3 hours of symptom onset (3); stroke patients treated within 90 minutes of onset of symptoms have the most improvement (4). Contacting emergency medical services (EMS) at the first sign of a stroke symptom allows for assessment of symptoms in the field, prenotification of the emergency department (ED), and activation of a stroke team. The steps reduce delays in receiving treatment (5). During the past decade, efforts have been made to reorganize prehospital systems of care, including EMS, to ensure that patients suspected to be experiencing a stroke arrive at a hospital equipped to provide timely assessment (3,6). In many localities, EMS protocols have been established to ensure that patients with possible stroke are transported to the nearest stroke center (if one is available) within an appropriate time frame for assessment and treatment (6). Despite these efforts, between 1993/94 and 1999, arrival times showed no significant increase in the percentage of stroke patients arriving at an ED within 2 hours and only a slight significant increase in the percentage arriving within 3 hours of symptom onset (7). Only 23% of patients admitted for any type of stroke arrived at the ED within 3 hours of symptom onset (4).

One barrier to timely care for ischemic stroke patients is either not identifying the symptom as a stroke symptom or not identifying the symptom as serious enough to seek immediate care (2,8,9). Community-based interventions have been developed to increase awareness of stroke symptoms and the need for emergency care for symptoms (10-13). Media campaigns have been implemented to increase knowledge of stroke symptoms and awareness of the need to seek urgent treatment by calling 9-1-1. Stroke symptom knowledge has increased over time (14), but this has not necessarily increased intention to call 9-1-1 (11) or use of 9-1-1 and EMS. Delay in seeking care has been largely unaffected (9).

We describe a media campaign for stroke awareness and the evaluation of the campaign's effect on awareness of stroke symptoms and the need for urgent care in Upstate New York. The purpose of the study was to develop, implement, and evaluate the impact of a stroke awareness media campaign by using a preintervention and postintervention design with a comparison region to control for temporal bias. We hypothesized that intention to call 9-1-1 to seek emergency care in response to stroke symptoms would increase more in the intervention region than in the comparison region. We also evaluated media campaign exposure and message recognition.

## Methods

### Setting

The intervention region consisted of Upstate New York counties Albany, Schenectady, and Rensselaer (2006 total population: 603,288) (15). The population was predominantly white, well educated, and employed (15). Orange County, the comparison region, which was matched for age, sex, and stroke rates, is south of the intervention region and is in a different media market. The population there (2006 total population: 376,392) was also predominantly white, well educated, and employed (15).

### Development and implementation of the intervention

Four focus groups were conducted with a total of 40 adults aged 30 years or older. Focus group participants were recruited using the Markette Research database of contacts as well as through referrals. Participants were contacted and screened for eligibility prior to the focus group. Participants were asked about their knowledge of stroke symptoms, the appropriate response to 4 stroke symptoms, and their sources of health information. Participants did not think that stroke symptoms required urgent action and did not think that there were time constraints for receiving effective treatment. Television was identified as a major source of health information. Participants reported using WebMD and pharmaceutical and insurance company Web sites for confirming or verifying health information they received from other sources. These findings guided the media campaign development.

During 2 of the 4 focus groups, participants responded to existing television and radio spots. Two television and 1 radio spot were well received and subsequently used in the campaign. New television spots were developed that emphasized the importance of timely arrival at the ED because none of the existing spots addressed time. These spots and other campaign materials emphasized that patients needed to arrive at the hospital within 1 hour of symptom onset to be eligible to receive the most effective treatment (4).

We used the FAST mnemonic to develop the new materials (F for Face drooping, A for Arm weakness, S for Speech slurred, and T for Time to call 9-1-1). Among patients with stroke or transient ischemic attack, the symptoms covered in the FAST mnemonic identified 89% of patients with stroke and 91% with ischemic stroke (16).

This stroke awareness media campaign consisted of 2 existing and 1 new 30-second, paid television spots that ran on broadcast and cable channels, a 60-second radio spot, public transit advertisements, community presentations by hospital stroke coordinators, table placards, pharmacy cards, and magnets (distributed during presentations and health fairs). The presentations were conducted at senior centers, worksites, and churches. The placards and other print materials were distributed at these sites and at health fairs, shopping malls, hospitals, and outpatient clinics. An example of a television spot can be viewed at http://www.health.state.ny.us/diseases/cardiovascular/stroke/.

The timing of the 3-phase campaign intervention design (Table 1) was based on when the new advertisements were available for dissemination and the availability of funding. Phase 1 was a low-intensity phase, defined by having a low gross rating point (GRP) (the percentage of the target population who saw the advertisement multiplied by the number of times the audience saw the advertisement during a specific time) that ran for 12 weeks during October through December 2006. Phase 2, the most intense phase, ran for 5 weeks in January and February 2007. Phase 3 was less intensive than Phase 2, running for 16 weeks from March through June 2007.

Campaign costs included all media purchases and production costs. The total cost for the television advertisements was $171,308; for the radio advertisements, $82,079; for transit advertisements, $37,512; and for print advertisements, $3,152. Print advertisements included 1,300 table placards, 11,000 pharmacy cards distributed to 161 pharmacies, and 480 magnets.

### Evaluation design

Survey data were collected before the media campaign began (baseline, July-September 2006) and during the media campaign's final, less-intensive phase (follow-up, March and April 2007). The matched design was implemented to control for potential temporal changes in the outcomes of interest and for demographic differences potentially associated with the outcomes. The evaluation consisted of a list-assisted, random-digit–dialed telephone survey. The institutional review board of the State University of New York at Albany approved the study.

### Sampling

Adults aged 30 years or older were randomly selected to participate. Sampling was proportionate to the population of each county. The total intervention sample (baseline n = 994 and follow-up n = 989) was 1,983. The total comparison sample (baseline n = 795 and follow-up n = 687) was 1,482.

### Survey instrument

The survey instrument consisted of questions about demographic characteristics, health care use, past EMS use, potential campaign exposure, and intended behavior in response to each of 4 stroke symptoms in oneself or another person. Demographic and health history questions were based on previously validated questions from national surveys (17,18). Questions about intention to call 9-1-1 for stroke symptoms were based on the Stroke Factor Survey (14) and Stroke Action Test (19). Media use and exposure questions were based on survey items from other studies (20,21). Questions asking about behavioral intentions in response to symptoms of stroke and to decoy symptoms are listed in the Appendix.

### Data collection

The survey was pilot-tested. The baseline survey was administered during July through September 2006. The follow-up data collection was during March and April 2007, after the intensive phase. It overlapped with Phase 3 of the campaign. The survey was administered by using standard Behavioral Risk Factor Surveillance System (BRFSS) protocols (18). The baseline survey response rate was 36% and the follow-up response rate was 35%, using the American Association for Public Opinion Research response rate 3 method (22).

### Data analysis

Data were analyzed by using Stata version 9 (StataCorp LP, College Station, Texas) and weighted by age, sex, and racial distribution of the population and to reflect the probability of being selected. Frequencies of descriptive variables were calculated by using unweighted data. Findings are reported with 95% confidence intervals (CIs), and significance assessed at the *P* ≤ .05 level. Sex, age, and race distributions of the sample were weighted to the comparison county. For each stroke symptom, the change between baseline and follow-up in the percentage of respondents who would call 9-1-1 was calculated and the difference between changes in the intervention and comparison regions was determined by using Stata's lincom procedure.

## Results

### Study sample

Demographic characteristics of the study samples from the comparison and intervention regions were similar at baseline and follow-up (Table 2). Fewer than 2% of respondents participated in both surveys.

### Multimedia campaign exposure

**Figure 1:**
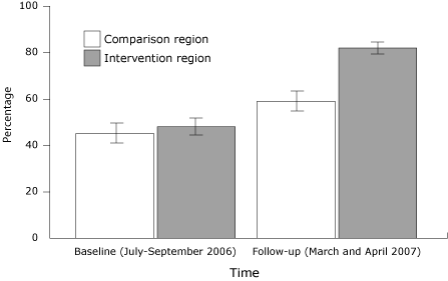
Proportion (with 95% confidence interval [CI] bars) of Upstate New York survey respondents who saw a bus or television advertisement or heard a radio advertisement about stroke symptoms. Comparison region was Orange County; intervention region was Albany, Schenectady, and Rensselaer counties.

At baseline, fewer than half of survey respondents reported having seen or heard any (television, transit, radio) stroke advertisements (Figure 1). Compared with baseline, at follow-up there was a 20 percentage point greater increase in the percentage of respondents who reported seeing or hearing stroke advertisements in the intervention region compared with the comparison region (95% CI, 12-28) (*P* < .001). At follow-up, among those in the intervention region who saw or heard an advertisement, 80% reported seeing a television advertisement compared with 50% who heard radio and 16% who saw transit advertisements.

**Table:** 

**Time**	**Comparison Region, Weighted Percentage (95% CI)**	**Intervention Region, Weighted Percentage (95% CI)**
Baseline (July-September 2006	45.2 (40.9-49.5)	48.1 (44.3-51.8)
Follow-up (March and April 2007)	59.1 (54.7-63.5)	82.1 (79.2-85.0)

**Figure 2:**
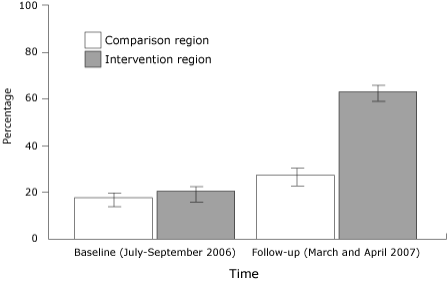
Proportion (with 95% confidence interval [CI] bars) of Upstate New York survey respondents reporting the stroke advertisement's message was to call 9-1-1, who saw a bus or television advertisement or heard a radio advertisement about stroke symptoms. Comparison region was Orange County; intervention region was Albany, Schenectady, and Rensselaer counties.

There was a greater increase in the percentages who reported that the advertisement's message was to call 9-1-1 from baseline to follow-up among respondents in the intervention region compared with respondents in the comparison region (Figure 2). Compared with baseline, at follow-up there was a 33 percentage point greater increase in the percentage of respondents who reported seeing or hearing stroke advertisements in the intervention region compared with the comparison region (95% CI, 26-40) (*P* < .001).

**Table:** 

**Time**	**Comparison Region, Weighted Percentage (95% CI)**	**Intervention Region, Weighted Percentage (95% CI)**
Baseline (July-September 2006	17.8 (14.7-20.9)	20.6 (17.5-23.6)
Follow-up (March and April 2007)	27.5 (23.5-31.5)	63.2 (59.6-66.8)

### Intention to call 9-1-1 for stroke symptoms

The baseline percentages of respondents reporting intention to call 9-1-1 for themselves or another person were similar among the intervention and the comparison regions (Table 3). At follow-up, the percentages reporting intention to call 9-1-1 were higher among the intervention region than the comparison region for all 4 stroke symptoms.

For all stroke symptoms at both times, the changes from baseline to follow-up in percentage reporting intention to call 9-1-1 were greater among the intervention region compared with the comparison region, and the differences between them were significant for most symptoms (Table 3). The largest increases in reported intention to call 9-1-1 were seen in response to inability to speak correctly in oneself and for an uneven face in another. The smallest difference in change between intervention and comparison regions was seen for inability to speak correctly in another. The proportion of respondents stating they would call 9-1-1 for any of the nonstroke (decoy) symptoms did not differ at baseline or follow-up, nor was there a difference over time between respondents living in the comparison and intervention regions.

## Discussion

These findings suggest that a multimedia campaign can increase the percentage of people who intend to call 9-1-1 in response to stroke symptoms in themselves or in another person. Intention to engage in a behavior is one of the best behavioral predictors (23). This increase in intention to call 9-1-1 was specific for the stroke symptoms and did not generalize to nonstroke symptoms. Most media campaigns to change health behaviors can expect to reach 50% to 90% of the target audience (24). Evidence that the new advertisements reached the audience is reflected in the large increase in people reporting that the stroke advertisement message was to call 9-1-1.

Some of the increase in awareness during the media campaign among in the intervention and the comparison regions may be due to media attention about a national figure (a US senator) who suffered a stroke in December 2006 and from media events during Stroke Awareness Month in May. Both regions have designated stroke centers that ran advertisements about their designation during the intervention period. The increase in awareness and seeing stroke advertisements in the comparison region confirms the importance of having a comparison group to account for temporal events or other naturally occurring public health-related activities in addition to the intervention. The significant increase in respondents from the intervention region who saw advertisements compared with respondents from the comparison region bolsters support for the effectiveness of the New York State Department of Health's multimedia campaign.

Similar to other studies, our study used television and radio, but also used print media and presentations. These likely helped media campaign exposure. Unlike most previous stroke awareness media campaigns, our study ascertained not just increased knowledge of stroke symptoms but reported intention to perform a targeted behavior. In addition, we used symptom questions that did not lead respondents; the questions did not indicate that the symptoms were specifically stroke symptoms. Some previous studies measured stroke symptom awareness by giving respondents a list of symptoms and asking them which are stroke symptoms (25), asked what they would do if they thought someone was having a stroke (10,11,13,25), and asked respondents to list stroke symptoms (12). Surveys that prompt respondents about stroke symptoms report inflated percentages of respondents with knowledge of stroke symptoms (26). Some previous studies also measured stroke symptoms that were not covered in our campaign (10,11,13,25,27).

Our study is similar to a study in Montana (11) in which television and radio advertisements promoted stroke symptom awareness and calling 9-1-1 for symptoms. Our study appears to have had a greater impact on the percentage of people who reported they would call 9-1-1 (11). The increase in the percentage of people who would call 9-1-1 if they identified symptoms in themselves was similar, but the Montana study did not find significant changes in the proportion of people who would call 9-1-1 if they witnessed someone else having a stroke (11). Our media intervention was 8 weeks longer (based on the number of weeks between the start of the intervention and the follow-up survey) but less intensive in terms of its television and radio advertisements and included more types of media in various outlets.

### Limitations

Our study findings are potentially limited by the low response rates. However, the response rates are similar to the New York State BRFSS survey response rate (39%) and the median BRFSS response rate (33%) for all states (28). It is not known whether response rates differed by demographic subgroups because the sampling strategy was not designed to determine these differences. Since the study population was primarily white and well educated, the findings are not generalizable to more diverse or less-educated populations. Another limitation resulted from the media campaign being delayed because of an underestimation of the time it took to produce the advertisements. The delay resulted in the need to conduct the follow-up survey and finalize the evaluation before the end of the grant period. Therefore, follow-up was conducted during the last, low-intensity campaign phase. The follow-up results may reflect very recent exposure to stroke advertisements. Results may have been lower had there been a period with no stroke advertisements before the follow-up assessment (12). There were additional advertisements from designated stroke centers that were advertising their designation, but this occurred in both the intervention and comparison regions.

### Summary

The greatest barrier to improving treatment of stroke is timely arrival of people with stroke symptoms at EDs that are capable of delivering timely stroke treatment. The results of this multimedia campaign, which emphasized 4 major stroke symptoms and the urgency of calling 9-1-1, suggest that the targeted audiences received this message. How long this message will be retained and whether the reported intention actually results in calling 9-1-1 if the need arises has yet to be determined.

Further research is needed to determine whether increased recognition and reporting of intention to seek emergency care by calling 9-1-1 results in an increase in calls to 9-1-1 and earlier arrival after stroke symptom onset, earlier treatment, and improved stroke outcomes. Regardless, these findings are encouraging and support increased efforts to implement multimedia campaigns for more sustained periods in wider media markets to maximize improvements in stroke outcomes. Because of the promising findings from this study, the New York State Department of Health is working with designated stroke centers across New York State to expand the campaign.

## Figures and Tables

**Table 1:** Advertising Campaign to Increase Awareness of Symptoms of Stroke, Upstate New York, 2006-2007

**Phase/Medium**	Symptom	Message	Duration, wks	No. of Times Advertisement Was Presented	GRP^a^
**Phase 1, October-December 2006**					
Television	Sudden arm or leg weakness, sudden trouble speaking, sudden trouble seeing	At the first warning sign take action immediately; call 9-1-1 immediately	12	123	101
**Phase 2, January and February 2007**					
Television	FAST^b^	Call 9-1-1, get to the hospital within 1 hour	5	1,558	1,900
Radio	FAST	Call 9-1-1, get to the hospital within 1 hour	2	520	608
**Phase 3, March-June 2007**					
Television	FAST	Call 9-1-1, get to the hospital within 1 hour	16	614	683
Radio	FAST	Call 9-1-1, get to the hospital within 1 hour	4	1,037	1,216
Transit	FAST	Call 9-1-1 at any of these signs of a stroke	8	36 bus shelters 68 bus sides, and 104 interior bus cards	50% of target population reached

a Gross rating point (GRP) is the percentage of the target population, referred to as rating point, who saw the advertisement (reach) multiplied by the number of times the audience saw the advertisement (frequency) during a specific time. A rating point is the size of an audience expressed as a percentage of the total potential audience.

b Symptoms covered by the FAST mnemonic: Facial droop, Arm weakness, Speech slurred, and Time to call 9-1-1.

**Table 2:** Description of Telephone Survey Sample in the Comparison and Intervention Regions,^a^ at Baseline and Follow-up,^b^ Upstate New York, 2006-2007

Characteristic	Comparison Region		Intervention Region	

	Baseline (n = 795)	Follow-up (n = 687)	Baseline (n = 994)	Follow-up (n = 989)
**Sex, n (%)**				
Male	290 (36.5)	261 (38.0)	345 (34.7)	391 (39.5)
Female	505 (63.5)	426 (62.0)	649 (65.3)	598 (60.5)
**Age, y, mean (SD)**	54 (14)	54 (14)	56 (15)	56 (14)
**Hispanic, n (%)**	43 (5.4)	39 (5.7)	17 (1.7)	25 (2.5)
**Race, n (%)**				
White	684 (87.6)	593 (88.5)	893 (90.9)	881 (89.8)
Black	53 (6.8)	44 (6.6)	53 (5.4)	59 (6.0)
Other	44 (5.6)	33 (4.9)	36 (3.7)	41 (4.2)
**Highest grade completed, n (%)**				
Less than high school diploma	75 (9.5)	48 (7.1)	92 (9.3)	64 (6.5)
High school diploma or general educational development certificate	181 (22.9)	155 (22.8)	216 (21.9)	207 (21.0)
Some college — associate's degree or technical program	229 (29.0)	191 (28.1)	265 (26.9)	263 (26.7)
Bachelor's degree	153 (19.4)	140 (20.6)	211 (21.4)	200 (20.3)
Graduate-level education or degree	152 (19.2)	147 (21.6)	203 (20.6)	252 (25.6)
**Employment status, n (%)**				
Employed	473 (59.7)	440 (64.1)	563 (56.6)	583 (59.1)
Not employed	116 (14.7)	91 (13.3)	123 (12.4)	136 (13.8)
Retired	203 (25.6)	155 (22.6)	308 (31.0)	268 (27.2)
**Health care coverage, n (%)**	764 (96.2)	655 (95.5)	943 (95.0)	957 (97.0)
**Answered baseline survey,^c^ n (%)**	—	11 (1.6)	—	18 (1.8)

Abbreviation: SD, standard deviation.

a Comparison region was Orange County; intervention region was Albany, Schenectady, and Rensselaer counties.

b Baseline survey administration, July through September 2006; follow-up survey, March and April 2007. Numbers may not equal sample total because of missing data.

c The question about taking the baseline survey was asked only on the follow-up survey.

**Table 3:** Percentage of Respondents Who Stated They Would Call 9-1-1 in Response to Stroke Symptoms in the Comparison and Intervention Regions,^a^ at Baseline and Follow-up,^b^ Upstate New York, 2006-2007

Symptom	Comparison Region		Intervention Region		Difference in Change^c^	

	Baseline % (95% CI)	Follow-up % (95% CI)	Baseline % (95% CI)	Follow-up % (95 % CI)	Difference (95% CI)	*P* Value
**Stroke symptom in self**						
Inability to speak correctly	54.7 (50.4-59.0)	59.3 (55.0-63.7)	54.3 (50.5-58.0)	71.3 (67.9-74.8)	12.5 (4.5-20.5)	.002
Sudden trouble seeing or double vision	24.7 (21.1-28.3)	24.1 (20.3-27.9)	22.7 (19.5-25.9)	31.3 (27.8-34.8)	9.1 (2.0-16.2)	.01
Sudden weakness in 1 arm	26.5 (22.8-30.1)	27.5 (23.5-31.5)	30.3 (26.8-33.8)	40.2 (36.4-43.9)	8.9 (1.5-16.3)	.02
**Stroke symptom in another**						
Inability to speak correctly	61.9 (57.7-66.1)	67.4 (63.2-71.5)	67.5 (64.0-71.0)	77.0 (73.8-80.2)	4.0 (-3.6 to 11.6)	.30
Sudden trouble seeing or double vision	30.8 (26.8-34.7)	28.1 (24.1-32.0)	29.0 (25.6-32.5)	34.4 (30.8-38.0)	8.1 (0.6-15.6)	.03
Uneven face^d^	64.3 (60.2-68.4)	65.3 (61.0-69.5)	64.9 (61.3-68.5)	77.9 (74.8-81.0)	12.1 (4.4-19.7)	.002
Sudden weakness in 1 arm	47.3 (43.0-51.6)	44.9 (40.4-49.4)	47.2 (43.4-51.0)	53.5 (49.7-57.3)	8.7 (0.5-17.0)	.04

Abbreviation: CI, confidence interval.

a Comparison region was Orange County; intervention region was Albany, Schenectady, and Rensselaer counties. Data were weighted by age, sex, and racial distribution of the population and to reflect the probability of being selected.

b Baseline survey administration, July through September 2006; follow-up survey, March and April 2007.

c Significance was calculated using a design-based *F* test. The Stata (StataCorp LP, College Station, Texas) lincom procedure was used to compute the change from baseline to follow-up in each group and compare the changes between groups and to assess the significance of the difference in change between groups.

d Uneven face was not asked in oneself because it was decided that this symptom would be difficult to self-assess and that it would be recognized only by someone else.

## Appendix. Survey Questions About Behavioral Interventions in Response to Symptoms of Stroke and to Decoy Symptoms, Upstate New York, 2006-2007

Respondent answered each question by choosing 1 of the following: 1) Wait, watch symptoms, and then decide what to do, 2) Call a family member or friend, 3) Call a doctor or nurse, 4) Call 9-1-1 or emergency medical services, 5) Other (specify), 6) Don't know, or 7) Refused.

### Stroke Symptom Questions

1. What would you do if YOU experienced an inability to speak correctly? For instance, people you were talking with could not understand you, they said you were slurring or using inappropriate words. Would you . . .
2. What would you do if YOU experienced sudden trouble seeing or double vision? Would you . . .
3. What would you do if YOU experienced sudden weakness in 1 arm? Would you . . .
4. What would you do if you saw or heard ANOTHER PERSON speak in a way that you could not understand? For instance, another person was slurring words or using inappropriate words. The person could not repeat a simple sentence. Would you . . .
5. What would you do if you heard ANOTHER PERSON complain about having sudden trouble seeing or double vision? Would you . . .
6. What would you do if you saw ANOTHER PERSON whose face looked uneven? It drooped on one side or they could not smile evenly. His or her smile didn't look like it usually does. Would you . . . (Face weakness/numbness in oneself was not asked because it was decided that this symptom would be difficult to self-assess and that it would be recognized only by someone else.)
7. What would you do if you heard ANOTHER PERSON say that he or she was suddenly feeling weak in 1 arm, or complain about a tingling sensation and numbness in the arm? Would you . . .

### Decoy Questions

1. What would you do if YOU experienced sharp back pain between your shoulder blades when you took a breath? Would you . . .
2. What would you do if YOU experienced stomach cramps? Would you . . .
3. What would you do if YOU experienced a temperature of 101 degrees or higher for 12 hours or more? Would you . . .
4. What would you do if you saw or heard ANOTHER PERSON had a temperature of 101 degrees or higher for 12 hours or more? Would you . . .
5. What would you do if YOU experienced pain or burning during urination? Would you . . .
6. What would you do if you heard ANOTHER PERSON complain about a sharp back pain between his or her shoulder blades when he or she took a breath? Would you . . .
